# Okinalysin, a Novel P-I Metalloproteinase from* Ovophis okinavensis*: Biological Properties and Effect on Vascular Endothelial Cells

**DOI:** 10.3390/toxins6092594

**Published:** 2014-08-25

**Authors:** Yumiko Komori, Eri Murakami, Kei-ichi Uchiya, Tunemasa Nonogaki, Toshiaki Nikai

**Affiliations:** 1 Department of Microbiology, Faculty of Pharmacy, Meijo University, 150 Yagotoyama, Tenpaku, Nagoya 468-8503, Japan; E-Mails: paku.hgrn-ed15@ezweb.ne.jp (E.M.); kuchiya@meijo-u.ac.jp (K.U.); nikai@meijo-u.ac.jp (T.N.); 2 College of Pharmacy, Kinjo Gakuin University, 2-1723 Omori, Moriyama, Nagoya 463-8521, Japan; E-Mail: tunenono@kinjo-u.ac.jp

**Keywords:** *Ovophis okinavensis* venom, hemorrhagic toxin, metalloproteinase, vascular endothelial cell, cytotoxicity

## Abstract

A novel hemorrhagic metalloproteinase, okinalysin, was isolated from the venom of *Ovophis okinavensis*. It possessed caseinolytic and hemorrhagic activities, and also hydrolyzed fibrinogen and collagen. These activities were inhibited by ethylenediaminetetraacetic acid (EDTA) but not by *p*-amidinophenyl methanesulfonyl fluoride hydrochloride (APMSF). The molecular mass of okinalysin was 22,202 Da measured by MALDI/TOF mass spectrometry. The primary structure of okinalysin was partially determined by Edman sequencing, and the putative zinc-binding domain HEXXHXXGXXH was found to be present in its structure. From these data, okinalysin is defined as a metalloproteinase belonging to a P-I class. The partial amino acid sequence of okinalysin was homologous to the *C*-terminus of MP 10, a putative metalloproteinase induced from transcriptome of the venom gland cDNA sequencing of *O. okinavensis*. Okinalysin possessed cytotoxic activity on cultured endothelial cells, and the *EC*_50_ on human pulmonary artery endothelial cells was determined to be 0.6 μg/mL. The histopathological study also showed that okinalysin causes the leakage of red blood cells and neutrophil infiltration. These results indicate that destruction of blood vessels by okinalysin is one of the main causes of hemorrhage.

## 1. Introduction

Among the various kinds of enzyme and protein existing in snake venoms, metalloproteinase (SVMP: snake venom metalloproteinase) is one of the most important components. The role of SVMPs in the pathologies associated with Viperidae envenomation has long been especially studied. Varieties of SVMPs were reported which cause symptoms such as hemorrhage, fibrinogenolysis, necrosis and apoptosis [1,2,3,4,5,6,7,8,9,10]. Fox and Serrano described the protein structural classification of SVMPs [11]; Class P-I has only a metalloproteinase domain, Class P-II consists of metalloproteinase and disintegrin domains, Class P-III is synthesized with metalloproteinase, disintegrin-like and cysteine-rich domains, and Class P-IV has the P-III domain structure and lectin-like domains. Venom gland cDNA sequencing studies indicated that these SVMPs were biosynthesized as latent precursor pro-proteinases [12,13]. In general, the hemorrhagic activity of SVMPs of Class P-I is less active than P-III SVMPs, because disintegrin-like domains and cysteine-rich domains are considered to have functions in interacting with cell surface or cell matrix [14].

In the southern islands of Japan, most snake envenomation is due to Okinawa habu (*Protobothrops flavoviridis*). The frequency of envenomation by Himehabu (*O. okinavensis*) is low because of the short venomous fangs and small content of venom. Since the average number of victims of Himehabu envenomation in a year is approximately 10, this venom has not been studied in detail. Aird *et al*. [15] analyzed the venom gland cDNA transcripts of *O. okinavensis* and showed that 95 venom-related proteins are included. The major venom constituents were serine-proteinases (93.1%) and the percentage of metalloproteinases was only 4.2%. In contrast, the dominant constituents of *P. flavoviridis* venom glands are phospholipase A_2_ (32.1%) and metalloproteinases (27.0%). Since *O. okinavensis* and *P. flavoviridis* have different feeding habits; the former mainly feeds on small frogs while the latter preys on mammals such as mice [16,17,18], the venom components necessary for predation might be different. For the reasons given above, hemorrhagic toxins in the venom of *O. okinavensis* have not been well studied. However, it is necessary to know the characteristics of the venom to provide better treatment for envenomation. In this paper, we report the isolation and biochemical characterization and the mechanism of hemorrhage of a novel hemorrhagic metalloproteinase from *O. okinavensis* venom.

## 2. Results and Discussion

### 2.1. Isolation and Properties

**Figure 1:**
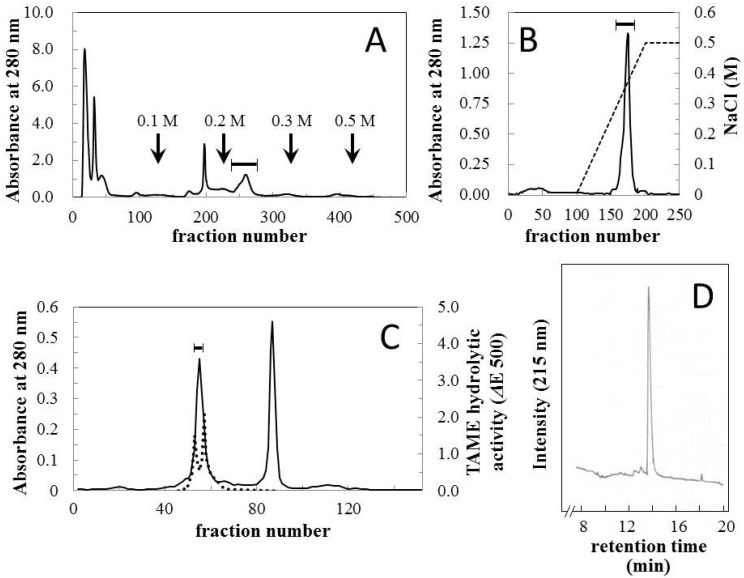
Isolation of okinalysin from *O. okinavensis* venom. (**A**) CM Sephadex C-50 column chromatography. Crude venom (500 mg) was applied to a column (1.5 × 45 cm) equilibrated with 10 mM Tris-HCl buffer (pH 7.5) containing 10 mM NaCl, and the salt concentration was increased stepwise. Fractions of 3.0 mL were collected at a flow rate of 13.5 mL/min; (**B**) CM Sephadex C-50 column chromatography. The hemorrhagic fraction indicated with a solid bar in (**A**) was re-chromatographed on the same column, and eluted with a linear gradient from 0.01–0.5 M NaCl; (**C**) HW-50 gel filtration. Fractions 170–177 of the second step chromatography (**B**) were concentrated and fractionated with a size-exclusion column (2.5 × 70 cm); (**D**) Reversed-phase HPLC profile of the final preparation of okinalysin.

**Figure 2:**
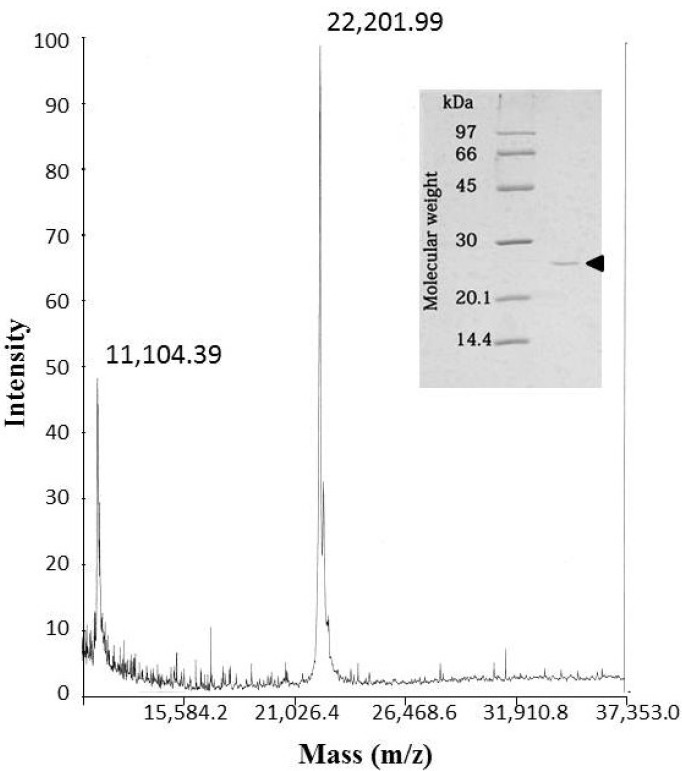
MALDI/TOF mass spectra of okinalysin from *O. okinavensis* venom. (insert) SDS-polyacrylamide gel electrophoresis.

Crude venom was fractionated using CM Sephadex C-50 column chromatography (Figure 1A) and relatively strong hemorrhagic activity was found in the fraction which was eluted with 0.2 M NaCl. The fraction was further purified using CM Sephadex C-50 and HW-50 columns (Figure 1B,C). The first fraction eluted from the HW-50 column possessed both hemorrhagic and arginine ester hydrolytic activities (Figure 1C). This fraction was then separated with ultrafiltration using Ultracel-30K, and a homologous hemorrhagic preparation was found to be present in the upper unit. The homogeneity of the final preparation was determined using reversed-phase HPLC (Figure 1D) and SDS-PAGE (Figure 2, insert), and it was named okinalysin.

The molecular mass of okinalysin determined by SDS-PAGE was found to be 24,500 Da, while the MALDI/TOF mass method gave a molecular weight of 22,202 Da (Figure 2).

### 2.2. Primary Structure

The enzymatically cleaved fragments of okinalysin by lysyl endopeptidase were subjected to Edman sequencing analysis. The fragments produced by autoproteolysis of okinalysin were also analyzed. The partially determined amino acid sequence contained a putative zinc-binding catalytic site, HEXXHXXGXXH, which is found in the metalloproteinase domain of SVMPs [11]. This result and the molecular weight of okinalysin indicate that this enzyme belongs to the P-I class of SVMPs.

**Figure 3:**
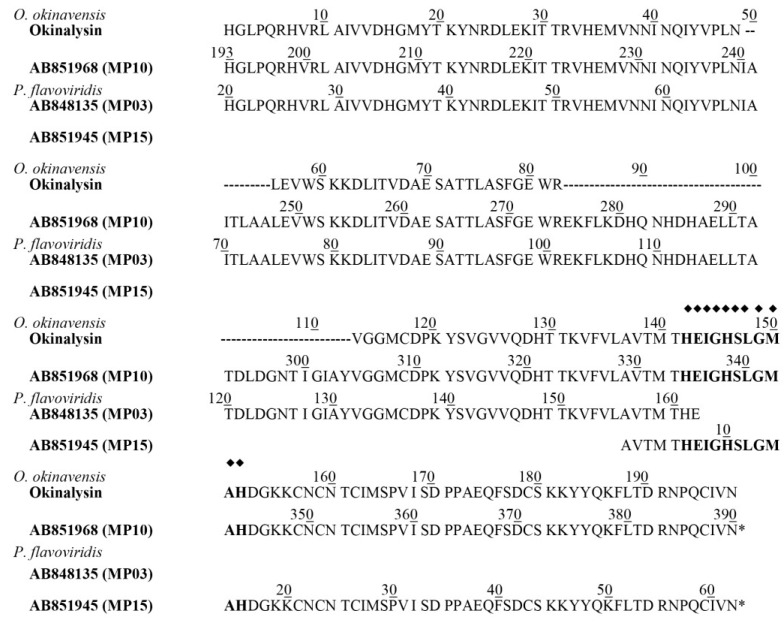
Comparison of partial amino acid sequence of okinalysin determined by direct sequencing (this study) with the predicted protein sequences obtained by the analysis of *O. okinavensis* and *P. flavoviridis* transcriptome. The protein sequence was aligned according to the position of MP 10 (DDBJ accession number of AB851968). The residues of okinalysin that were not determined by the direct sequencing were indicated by (-). The sequence of MP 10 was obtained from *O. okinavensis* transcriptome, and MP 03 (AB848135) and MP 15 (AB851945) were from *P. flavoviridis* transcriptome. The putative zinc-binding site is indicated by bold characters with (◆).

Recently, the results of venom gland cDNA sequencing of *O. okinavensis* and *P. flavoviridis* have been reported [15], and indicate that the *O. okinavensis* transcriptome included seven P-II metalloproteinases (MPs) and three P-III MPs as the transcripts. Among these sequences of MPs, the putative protein (MP 10) from the mRNA (DDBJ accession number-AB851968) was most homologous to the partial amino acid sequence of okinalysin (Figure 3). MP 10 is composed of 389 amino acids, and the theoretical molecular weight of peptide chain from His(193) to Asn(390) of MP 10 is calculated to be 22,198.34 Da. Since this molecular mass is almost identical to the molecular mass of okinalysin (22,201.99 Da) obtained by MALDI-TOF mass spectra, MP 10 probably consists of a pro-domain and a metalloproteinase domain (okinalysin).

Among 12 P-II metalloproteinase transcripts included in *P. flavoviridis* transcriptome, MP 03 (mRNA; DDBJ accession number-AB848135) and MP 15 (mRNA; DDBJ accession number-AB851945) also contained a similar sequence to okinalysin. In the sequence of MP 03, the peptide from His(20) to its *C*-terminus Glu is homologous to *N*-terminus 143 amino acid residues of okinalysin, and the sequence of MP 15 coincided with the *C*-terminal 62 amino acid residues of okinalysin (Figure 3). It is interesting that the enzymes found in the Ovophis and Protobothrops venoms have the same partial structure. *O. okinavensis* and *P. flavoviridis* were previously classified into a same genus *Trimeresurus*, but it is now reclassified into a different genus. However, there may be a similarity between their genes.

### 2.3. Enzyme Activities and Pharmacological Activities

Proteolytic activity of okinalysin was measured with or without inhibitors such as EDTA and *p*-amidinophenyl methanesulfonyl fluoride hydrochloride (APMSF). In the absence of these inhibitors, casein hydrolyzing activities of crude venom and okinalysin were determined to be 0.23 and 0.37 units/mg, respectively. The casein hydrolyzing activity of okinalysin was strongly inhibited by EDTA, while APMSF did not affect the activity. To avoid the effect of trace of serine-proteinase which might exist in the purified okinalysin preparation, all the enzyme and pharmacological assays described below were performed in the presence of APMSF at a final concentration of 0.5 mM.

Proteolytic specificity of okinalysin was examined with oxidized insulin B chain as a substrate, and the digested fragments were analyzed. The cleavage points of insulin B chain were determined to be His(5)-Leu(6), Ala(14)-Leu(15) and Tyr(16)-Leu(17), and these X-Leu positions are similar to the hydrolytic points by other SVMPs [19,20,21,22].

**Figure 4:**
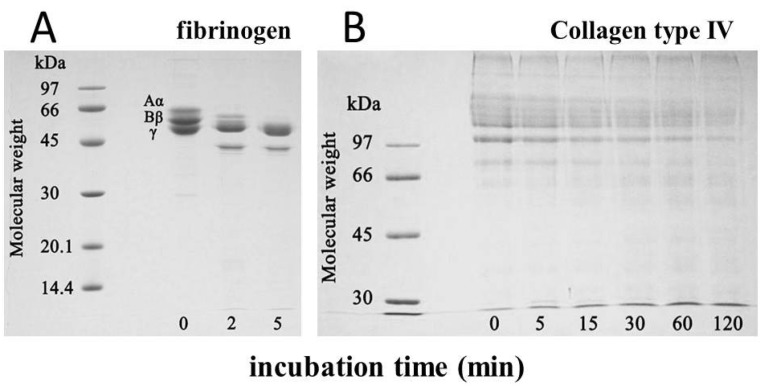
Hydrolytic activity of purified okinalysin on (**A**) bovine fibrinogen and (**B**) collagen type IV. Aα, Bβ, γ, denote the chains of fibrinogen.

The minimum hemorrhagic dose of okinalysin measured by subcutaneous injection was 6.6 µg/mouse. Hemorrhagic activity was completely inhibited by EDTA, and it was also lost after the incubation for 10 min at 70 °C. When bovine fibrinogen was incubated with okinalysin at a molar ratio of one to one, Aα and Bβ chains of fibrinogen were immediately hydrolyzed (Figure 4A). Okinalysin also possessed hydrolytic activity on collagen type IV (Figure 4B). These data indicate that proteolytic okinalysin participates in the destruction of the structurally important component of blood vessels, and disturbs hemostasis.

### 2.4. Toxicity Test on Cultured Cells

**Figure 5:**
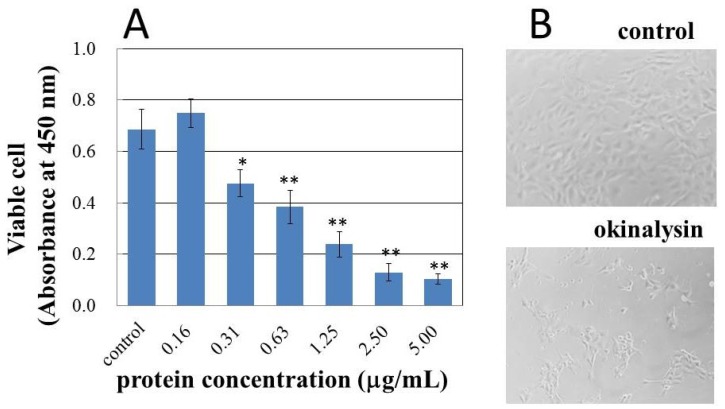
Cytotoxic effect of okinalysin on cultured human pulmonary artery endothelial cells (HPAEC). (**A**) Okinalysin solution in sterilized saline was added at various concentrations, and after 24 h, viable cells were counted by the colorimetric method. The results shown represent the average of five experiments. * *p* < 0.005, ** *p* < 0.001 compared to the control; (**B**) Phase-contrast micrographs (×100) of HPAEC control (upper) and cells incubated with okinalysin for 24 h at a final concentration of 5.0 μg/mL (lower).

Cultured human pulmonary artery endothelial cells (HPAEC) were used to estimate the effect of okinalysin on blood vessels. Figure 5A shows the changes in viable cell number after incubation with samples for 24 h. Compared with control cells, viable HPAEC clearly decreased, and only 15% of cells were alive after treatment with a final concentration of 5.0 μg/mL, and the *EC*_50_ on HPAEC was determined to be 0.6 μg/mL. The cytotoxic effect was also observed under phase-contrast microscope (Figure 5B). In the presence of okinalysin, decreases in adherent cells and changes in cell morphology were observed. The study of cytotoxicity using hemorrhagic metalloproteinase, rubelysin (HT-2) [3] and non-hemorrhagic rubelase indicated that the effect of non-hemorrhagic metalloproteinase was relatively weak [23]. When human umbilical vein endothelial cells (HUVEC) and HPAEC were used, rubelysin at concentrations of 1.25–5.0 μg/mL clearly induced cell death. While non-hemorrhagic rubelase possessed slight cytotoxicity at a concentration of 5.0 μg/mL, a more remarkable difference in cytotoxic effect was observed when aortic smooth muscle cells were used, and rubelase did not affect the cell viability. As indicated in Figure 5A, the cytotoxic effect of okinalysin on HPAEC at concentrations of 0.31–5.0 μg/mL is comparable to rubelysin. These results indicate that hemorrhagic metalloproteinases might affect endothelial cells and induce destruction of the vascular wall to cause hemorrhage. Further experiments using other hemorrhagic and non-hemorrhagic SVMPs are necessary to clarify these points.

### 2.5. Histopathological Study

**Figure 6:**
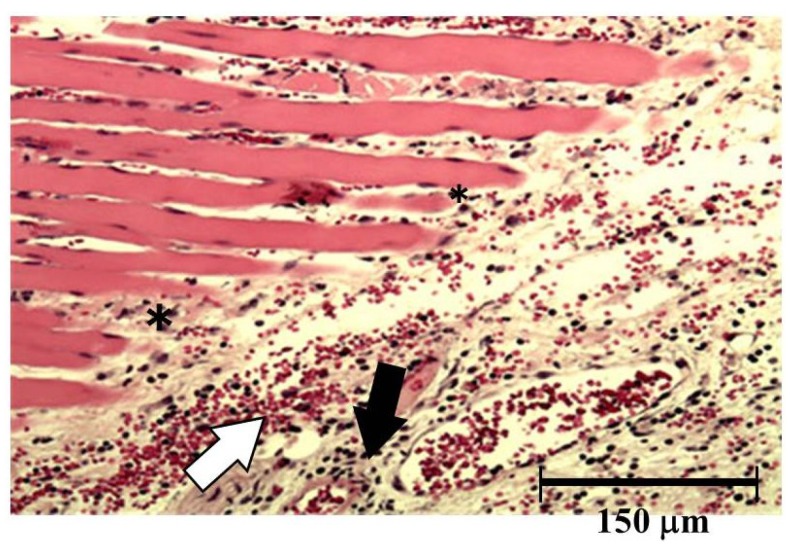
Light micrograph of muscle from the thigh of mice. Okinalysin (0.17 mg) was intramuscularly injected. White arrow: the emigration of red blood cells; Black arrow: neutrophil infiltrations; *: destruction of muscular fiber.

Both hemorrhage and permeation of neutrophil to the tissue were observed after injection of okinalysin into mice thigh (Figure 6). Destruction of muscular fiber also occurred 24 h after injection. However, these phenomena were relatively mild compared to metalloproteinases in other viperidae venoms such as *P. flavoviridis* and *Gloydius blomhoffii*, which possess strong hemorrhagic activity with a dose of 0.01–0.1 μg/mouse.

## 3. Experimental Section

Lyophilized crude venom of *Ovophis okinavensis* was purchased from The Japan Snake Institute (Gunma, Japan). CM Sephadex C-50 was obtained from GE healthcare (Tokyo, Japan), TOYOPEARL™ HW-50 was from Tosoh Co., Ltd. (Tokyo, Japan), and Amicon Ultra centrifugal filters: Ultracel-30K was the product of Merck Millipore Ltd. (Darmstadt, Germany). Sinapinic acid and casein were supplied by Nacalai tesque (Kyoto, Japan). Tosyl-L-arginine methyl ester was obtained from Peptide Institute Inc. (Osaka, Japan). Fibrinogen and oxidized insulin B chain were purchased from Sigma Chemical Co. (Perth, Australia), and collagen type IV from bovine lens was obtained from Nitta Gelatin Inc. (Osaka, Japan). *p*-Amidinophenyl methanesulfonyl fluoride hydrochloride (APMSF) and lysyl-endopeptidase were purchased from Wako Pure Chemical Industries, Ltd. (Osaka, Japan). Cryo-preserved human pulmonary artery endothelial cells (HPAEC) and their respective cell culture medium were obtained from Kurabo (Osaka, Japan). Cell counting kit-8™ was supplied by Dojindo laboratories (Kumamoto, Japan). Other chemicals were of analytical grade from commercial sources. All experiments involving the use of animals were carried out in compliance with the guidelines for animal experiments of Faculty of Pharmacy, Meijo University.

### 3.1. Isolation and Biochemical Properties

Okinalysin was isolated from crude venom by CM Sephadex C-50 cation-exchange column chromatography, HW-50 gel filtration and ultrafiltration using Ultracel-30K. The molecular weight was determined by SDS-polyacrylamide gel electrophoresis and MALDI-TOF mass spectrometry using Voyager™ Workstation (AB Sciex, Framingham, MA, USA). HPLC-purified and lyophilized okinalysin was dissolved in 0.1% acetonitrile, and mixed with equal amount of matrix (3,5-dimethoxy-4-hydroxycinammic acid dissolved in 70% acetonitrile containing 0.2% trifluoroacetic acid). The mixture was then applied onto the sample plate, and the system was operated in the linear mode according to fifth version of the operating manual.

### 3.2. Determination of Partial Structure

Okinalysin was enzymatically digested with lysyl endopeptidase. The digested fragments were also obtained by autoproteolysis, which occurs when okinalysin is incubated in 10 mM Tris-HCl buffer (pH 7.5) containing 10 mM NaCl at 37 °C for 23 h. The fragments were analyzed by the Edman degradation method using Applied Biosystems 491 protein sequencer and Model 610A PTH analyzer (Carlsbad, CA, USA) in accordance with the manufacturer’s instructions.

### 3.3. Enzyme Activities and Pharmacological Activities

Proteolytic activity was measured by the method of Murata *et al.* [24] using casein as the substrate, and arginine ester hydrolytic activity by the method of Roberts [25]. Fibrinogenolytic activity and collagen-hydrolytic activity were determined by the method of Ouyang and Teng [26]. Hemorrhagic activity was measured by the method of Bjarnason and Tu [27].

### 3.4. Toxicity Test on Cultured Cells

Frozen human pulmonary artery endothelial cells (HPAEC) were cultured and maintained in the appropriate medium according to the method of the supplier’s instructions. For bioassays, cells were seeded at a density of 1.5 × 10^4^ cells/well in 0.1 mL of medium in 96-multiwell plates. Samples were diluted in sterilized saline and then added to the cells. After 24 h, cell densities were determined by the colorimetric method using a cell counting kit-8 that was based on the tetrazolium salt/formazan system [28]. Cell-damage was also observed under a phase-contrast microscope (Olympus, Tokyo, Japan).

### 3.5. Histopathological Study

Histopathological study was performed by intramuscular injection of sample solution into the medial aspect of the thigh muscle of ddY strain white mice. The mice were sacrificed by ether-inhalation 24 h after injection. Tissue samples were immediately fixed in 10% neutral buffered formalin for 24 h at room temperature. The tissue was then washed for 4 h in running water, dehydrated in an autotechnicon, and stained with hematoxylin and eosin for observation under light microscope.

## 4. Conclusions

Okinalysin, a novel P-I class metalloproteinase, was isolated and the biological activities were examined. The existence of this proteinase had been proven at a gene level [15], and this study has shown biological activities and pathogenicity. Similarly to other hemorrhagic SVMPs, the structure of okinalysin contains a zinc-binding domain, HEXXHXXGXXH, and this proteinase possessed proteolytic activity on fibrinogen and type IV collagen. It also injured cultivated artery endothelial cells. 

Aird *et al*. [15] described that the major contents of *O. okinavensis* venom were not metalloproteinases but serine-proteinases. In fact, various serine-proteinase fractions had been obtained during the purification process, therefore, the main symptoms of *O. okinavensis* envenomation may be blood coagulation disorder, edema and hypotension caused by serine-proteinase. A small amount of hemorrhagic metalloproteinase in *O. okinavensis* venom may not possess severe effect alone; however, the blood coagulation disorder possibly increases hemorrhage when metalloproteinase coexists with serine-proteinase in crude venom.

When the results of the cytotoxicity study using cultivated cells are examined together with the experimental results of rubelase and rubelysin previously reported, it seems that the results of the cytotoxicity study well reflect the effect of snake venom hemorrhagic metalloproteinase. Since there are now cases when animal experiments are difficult to carry out from a point of view of the prevention of cruelty to animals, this method may become very useful for studying hemorrhage in the future. It is necessary to establish a method of cytotoxicity study using various hemorrhagic or non-hemorrhagic SVMPs.
